# Heme oxygenase-1 is dispensable for the anti-inflammatory activity of intravenous immunoglobulin

**DOI:** 10.1038/srep19592

**Published:** 2016-01-22

**Authors:** Caroline Galeotti, Pushpa Hegde, Mrinmoy Das, Emmanuel Stephen-Victor, Fernando Canale, Marcos Muñoz, Varun K. Sharma, Jordan D. Dimitrov, Srini V. Kaveri, Jagadeesh Bayry

**Affiliations:** 1 Institut National de la Santé et de la Recherche Médicale Unité 1138, Paris, F-75006, France; 2 Sorbonne Universités, UPMC Univ Paris 06, UMR S 1138, Paris, F-75006, France; 3 Centre de Recherche des Cordeliers, Equipe - Immunopathology and therapeutic immunointervention, Paris, F-75006, France; 4 Department of Pediatric Rheumatology, National Referral Centre of Auto-inflammatory Diseases, CHU de Bicêtre, le Kremlin Bicêtre, F-94270, France; 5 Université Paris Descartes, Sorbonne Paris Cité, UMR S 1138, Paris, F-75006, France; 6 International Associated Laboratory IMPACT (Institut National de la Santé et de la Recherche Médicale, France - Indian council of Medical Research, India), National Institute of Immunohaematology, Mumbai, 400012, India

## Abstract

Intravenous immunoglobulin G (IVIG) is used in the therapy of various autoimmune and inflammatory conditions. The mechanisms by which IVIG exerts anti-inflammatory effects are not completely understood. IVIG interacts with numerous components of the immune system including dendritic cells, macrophages, T and B cells and modulate their functions. Recent studies have reported that heme oxygenase-1 (HO-1) pathway plays an important role in the regulation of inflammatory response in several pathologies. Several therapeutic agents exert anti-inflammatory effects via induction of HO-1. Therefore, we aimed at exploring if anti-inflammatory effects of IVIG are mediated via HO-1 pathway. Confirming the previous reports, we report that IVIG exerts anti-inflammatory effects on innate cells as shown by the inhibitory effects on IL-6 and nitric oxide production and confers protection in experimental autoimmune encephalomyelitis (EAE) model. However, these effects were not associated with an induction of HO-1 either in innate cells such as monocytes, dendritic cells and macrophages or in the kidneys and liver of IVIG-treated EAE mice. Also, inhibition of endogenous HO-1 did not modify anti-inflammatory effects of IVIG. These results thus indicate that IVIG exerts anti-inflammatory effects independent of HO-1 pathway.

Initially used as replacement therapy in immune deficiencies, IVIG is also widely used for the treatment of a number of autoimmune and systemic inflammatory diseases^1^,^2^,^3^,^4^,^5^. Despite its therapeutic use for more than three decades, the precise mechanism by which IVIG exerts its beneficial effect is not fully understood. Exploration of mechanisms of IVIG is useful to define the dosage, to identify an appropriate window and duration of treatment, and to delineate biomarkers of therapeutic response. IVIG interacts with numerous components of the immune system including dendritic cells (DCs), macrophages, T and B cells and modulate their functions^6^,^7^,^8^,^9^,^10^,^11^,^12^,^13^,^14^,^15^,^16^,^17^,^18^,^19^,^20^,^21^. These mechanisms of IVIG also reflect the functions of circulating IgG in the maintenance of immune homeostasis.

Recent studies in various experimental models such as sepsis, cardiovascular pathologies, experimental autoimmune encephalomyelitis (EAE) and transplantation, and infection models such as *Mycobacterium tuberculosis* have highlighted the biological significance of heme oxygenase-1 (HO-1) enzymatic pathway and the reactive products of this pathway in regulating the inflammation and in the adaptation of the pathogens to the host microenvironment^22^,^23^,^24^,^25^,^26^,^27^,^28^. HO-1 catalyzes the degradation of heme, resulting in the liberation of equimolar amounts of iron, carbon monoxide (CO) and biliverdin. Biliverdin is subsequently converted to bilirubin by biliverdin reductase. Congenital defects in HO-1 expression in mice and human are associated with systemic inflammation^29^. HO-1 inhibits ovalbumin-induced airway inflammation by enhancing the biological activity of regulatory T cells (Tregs) in an IL-10-dependent manner^30^. Nevertheless, development, maintenance and the functions of Tregs under physiological conditions are not dependent on the activity of HO-1^31^.

CO and biliverdin have potent anti-inflammatory, anti-proliferative, anti-apoptotic, and antioxidant activities and exert their effects on many cell types, including cells of the immune system^32^. CO suppresses the pro-inflammatory response and promotes the anti-inflammatory programs of macrophages, DCs and monocytes^33^,^34^. Thus, either overexpression of HO-1 in innate cells or exposure to CO leads to inhibition of pro-inflammatory cytokines and enhancement of IL-10. CO also inhibits the lipopolysaccharide (LPS)-mediated maturation of DCs^35^,^36^.

Thus, in view of the common anti-inflammatory role exerted by both HO-1 and IVIG, we investigated if mechanisms of action of IVIG both *in vitro* and *in vivo* implicate HO-1 pathway.

## Results

### Anti-inflammatory effects of IVIG on human monocytes are not associated with induction of HO-1

**Figure 1:**
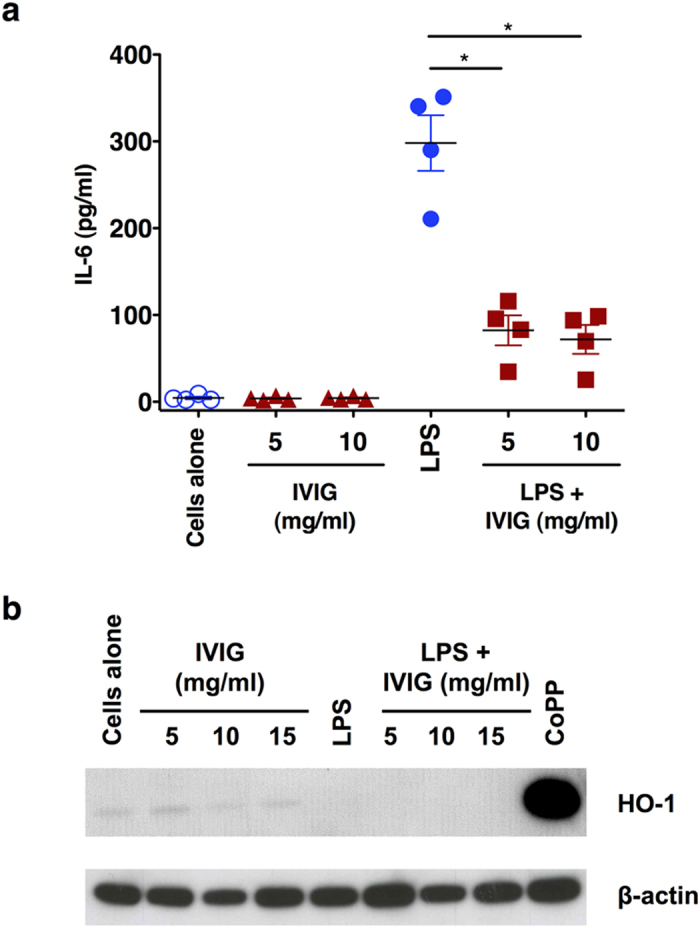
Anti-inflammatory effects of IVIG on human monocytes are not associated with induction of HO-1. (**a**) IVIG suppresses LPS-induced IL-6 production in human monocytes. Human peripheral blood monocytes were cultured in RPMI-1640 medium with 10% fetal calf serum either alone (cells alone) or with IVIG (5 and 10 mg/ml) for 24 hours. In some conditions, after 24 hours of culture, monocytes were exposed to LPS for additional 24 hours. IL-6 in the culture supernatants was measured by ELISA (n = 4). *p < 0.05, One-way ANOVA (**b**) Expression of HO-1 in human monocytes treated with IVIG (5, 10 or 15 mg/ml) alone or with LPS during last 24 hours of culture. CoPP was used as a positive control to induce HO-1. Images have been cropped for presentation and full-size blots are presented in Supplementary Figure S2.

It is known that IVIG exerts anti-inflammatory effects on innate cells such as monocytes, DCs and macrophages leading to suppression of inflammatory cytokines^8^,^37^,^38^,^39^. By analysing the production of IL-6, we first confirmed the anti-inflammatory action of IVIG. Unstimulated monocytes produced insignificant amount of IL-6. However, upon stimulation with LPS, a TLR4-agonist, monocytes produced large amounts of IL-6. Importantly, IVIG significantly reduced the production of IL-6, thus validating the anti-inflammatory effects of IVIG (Fig. 1a). The inhibition however was not dependant on the dose of IVIG.

We then examined the effect of IVIG on the expression of HO-1. Untreated monocytes expressed marginal amount of HO-1 and was not modified by IVIG. Even under inflammatory conditions, IVIG failed to induce the expression of HO-1 (Fig. 1b) in all tested concentrations. The lack of expression of HO-1 in IVIG-treated monocytes was not due to technical errors or non-functioning of HO-1-detecting antibodies as treatment of monocytes with cobalt protoporphyrin IX (CoPP), an inductor of HO-1, strongly induced HO-1. These results thus suggest that anti-inflammatory effects of IVIG on monocytes are independent of HO-1 pathway.

### Inability of IVIG to induce HO-1 in dendritic cells and macrophages

**Figure 2:**
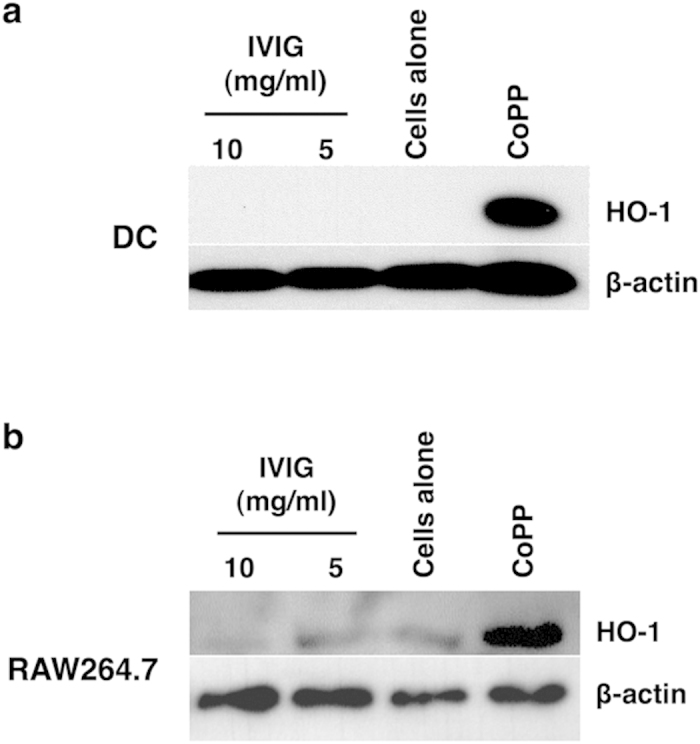
IVIG lacks the capacity to induce HO-1 in dendritic cells and macrophages. (**a**) Human peripheral blood monocyte-derived DCs or **(b)** RAW264.7 macrophages were cultured in the medium alone or with IVIG (5 and 10 mg/ml) for 24 hours. Expression of HO-1 was detected by western blot. CoPP was used as a positive control to induce HO-1. Images have been cropped for presentation.

We investigated the effect of IVIG on the expression of HO-1 in other innate cells. Consistent with the results obtained with monocytes, IVIG did not induce HO-1 both in monocyte-derived human DCs as well as RAW264.7 macrophage cell line (Fig. 2a,b). CoPP, the positive control, induced HO-1 in both the cell types. These results thus indicate that inability of IVIG to induce HO-1 is not restricted to particular innate cell.

### Inhibition of endogenous HO-1 is not coupled with reduced functioning of IVIG

**Figure 3:**
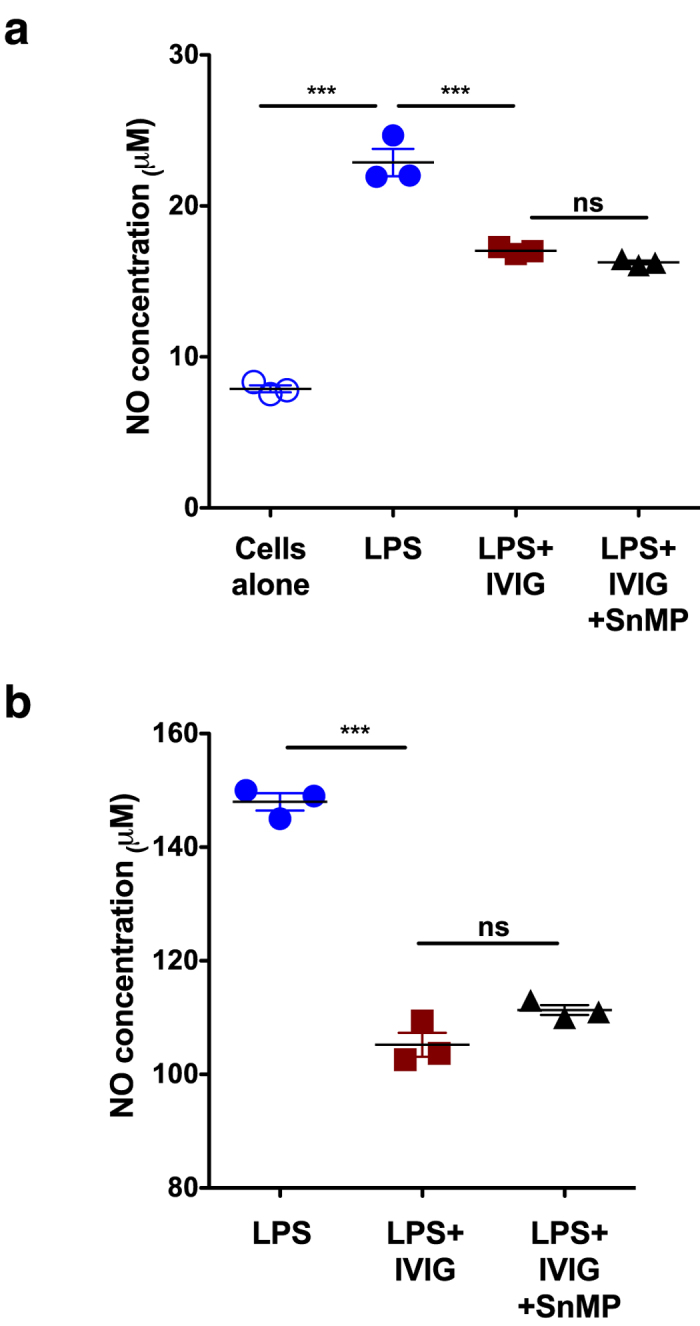
Inhibition of endogenous HO-1 is not associated with loss of anti-inflammatory action of IVIG. (**a**) Peritoneal macrophages from C57Bl/6J mice (n = 3) or (**b**) RAW264.7 macrophages (n = 3) were treated with IVIG (10 mg/ml) for 24 hours. They were then exposed to either LPS alone or LPS and SnMP for additional 24 hours. Production of NO was evaluated by Griess method. ***p < 0.001, One-way ANOVA and ns, not significant.

As innate cells express basal level of HO-1 (Fig. 1b, 2b), we wondered whether inhibition of this endogenous HO-1 is associated with reduced anti-inflammatory action of IVIG. Peritoneal macrophages from C57BL/6J mice and RAW264.7 cells were treated with IVIG for 24 hours and followed by stimulation with LPS. The activation of macrophages by LPS leads to oxidation of L-arginine via nitric oxide synthase and produce nitric oxide (NO). As shown in Fig. 3a,b, IVIG significantly inhibited LPS-induced NO production. However, treatment of cells with tin-mesoporphyrin (SnMP) to suppress enzyme activity of the endogenous HO-1 did not inhibit anti-inflammatory effects of IVIG on NO production (Fig. 3a,b).

### The protective effect of IVIG in experimental autoimmune encephalomyelitis is independent of induction of HO-1 *in vivo*

**Figure 4:**
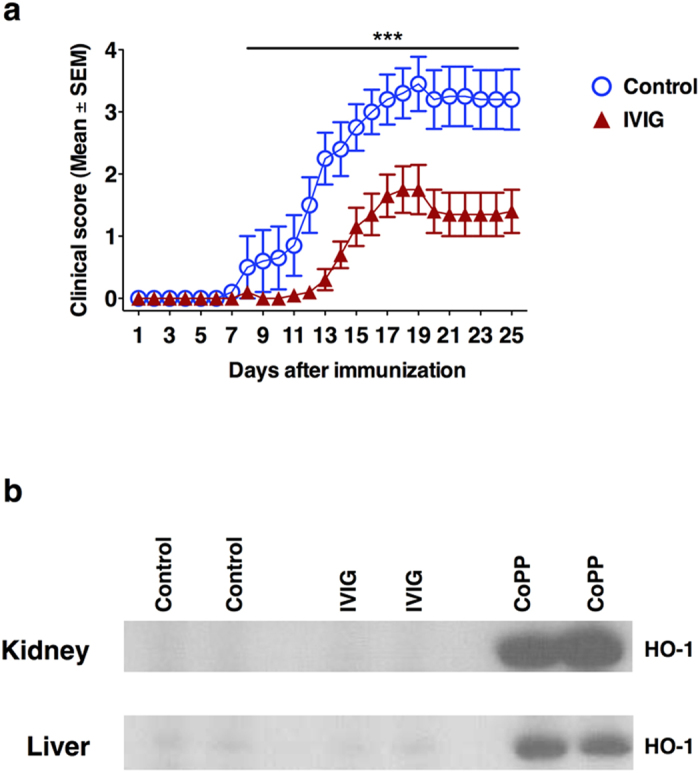
The protective effect of IVIG in EAE is independent of induction of HO-1 *in vivo*. (**a**) Treatment of mice (n = 10) with IVIG significantly delays the onset of EAE and the severity of the disease in C57BL/6J mice. Control mice (n = 10) received 0.2 M glycine while IVIG group received 0.8 g/kg IVIG daily from day 0 to 19. Mean clinical scores (±SEM) are presented. ***p < 0.001, two-way ANOVA with Bonferroni post-t-test. (**b**) Expression of HO-1 in kidney and liver of IVIG-treated EAE mice. The mice treated with CoPP were used as a positive control. Images have been cropped for presentation.

In order to validate the non-involvement of HO-1 pathway in IVIG-mediated anti-inflammatory effects *in vivo*, we resorted to EAE model. EAE was induced in C57BL/6J mice using MOG_35–55._ Confirming the previous reports, treatment of mice with IVIG significantly delayed the onset of EAE and the severity of the disease (Fig. 4a)^40^,^41^,^42^. However, consistent with *in vitro* results, this protection was not associated with an induction of HO-1 irrespective of the organs examined (liver and kidney) (Fig. 4b). Western blot analysis of lungs and spleen also showed same results. Naive mice injected with CoPP (20 mg/kg) were used as positive control for the expression of HO-1. In fact, expression of HO-1 was confirmed in the liver and kidneys of these mice 24 hours following CoPP injection (Fig. 4b).

## Discussion

It was suggested that HO-1 functions as a “therapeutic funnel”^32^. Several reports in experimental models have recently demonstrated that HO-1 pathway and its products could be used for the prevention or treatment of immune-mediated disorders. These protective effects are mediated by multiple functions of HO-1 that include immunosuppression, anti-inflammatory, anti-apoptosis and anti-oxidant effects. The anti-inflammatory mechanisms of HO-1 are mainly via modulation of activation of immune cells including antigen presenting cells and lymphocytes^32^. In fact, IVIG has been demonstrated to modulate the functions of both innate cells and T cells. Thus, IVIG was reported to suppress the activation of DCs, macrophages and monocytes and secretion of inflammatory cytokines while enhancing anti-inflammatory mediators like IL-10 and IL-1ra. In addition, IVIG also inhibits APC-mediated effector T cell activation, proliferation. Recent reports further demonstrate that IVIG inhibits Th1 and Th17 subsets, which are pathogenic in various autoimmune and inflammatory diseases, and reciprocally expands Tregs^40^,^41^,^42^,^43^,^44^,^45^,^46^,^47^,^48^. Importantly, HO-1 appears to be required for the action of several therapeutic molecules. For example, rapamycin appears not to exert its anti-proliferative effects on smooth muscle cells unless HO-1 is present^49^. All these different lines of evidence underscore the importance of dissection of HO-1 pathway in the anti-inflammatory effects of IVIG.

Previous reports have shown that innate cells express HO-1 in steady state and inhibit toll-like receptor-mediated (such as LPS) activation and secretion of pro-inflammatory cytokines^35^,^36^,^50^. Although, the expression of HO-1 in monocytes, DC and RAW264.7 was not prominent, we could detect basal expression of protein by western blot. However, IVIG did not modulate the basal expression of HO-1. Thus, suppression of LPS-mediated IL-6 and NO production by IVIG were independent of HO-1 pathway. It could be argued that IVIG-mediated suppression of IL-6 and NO might be due to passive neutralization of LPS by antibodies as we pre-treated the innate cells with IVIG before stimulation with LPS. However, as analyzed by the expression of CD80 and CD86, IVIG-mediated anti-inflammatory effects was well-preserved in the monocytes even if cells were stimulated with LPS followed by treatment with IVIG (Supplementary Fig. S1), thus ruling out passive neutralization of LPS as a mechanism of anti-inflammatory effect of IVIG. As activation stimuli such as LPS were reported to inhibit the expression of HO-1^35^, we opted for examining if IVIG-preconditioning results in HO-1 expression in innate cells, which in turn inhibits LPS-mediated activation of cells. Our data however clearly demonstrates that IVIG or otherwise, normal circulating antibodies lack the capacity to induce HO-1 and even upon inhibition of endogenous HO-1, IVIG-mediated anti-inflammatory effects were not altered.

Tregs play an important role in the prevention of autoimmune and inflammatory responses^51^. Initial reports have indicated that HO-1 in Tregs is critical for their immune suppression functions^52^. However, subsequent reports have contradicted this data and showed that HO-1 expression in Tregs is not key for immunoregulatory functions of these cells both in human and mice^31^,^53^. As stimuli from DCs are crucial in Treg expansion, subsequent report showed that HO-1 expression in DCs mediated suppressive functions of Tregs^54^. Despite induction of DC-mediated Treg expansion by IVIG as reported recently by us and others, we could not observe induction of HO-1 in DCs by IVIG suggesting that IVIG targets different pathways for the expansion of Tregs. In fact, several lines of evidence suggest that IVIG could expand Tregs via numerous mechanisms, which might act either independent or inter-linked^18^,^19^,^40^,^42^,^48^. The modulation of DC functions upon recognition of IVIG via DC-SIGN or DICER constitutes a major event^40^,^48^. This interaction leads to expression of COX-2-dependent PGE2 production in DCs, which in turn expands Tregs. The role of PGE2 in IVIG-mediated Treg expansion was documented both *in vivo* in animal models and in autoimmune patients treated with IVIG^40^,^45^.

Experimental models have shown that HO-1 pathway inhibits pathogenic T cell responses. EAE has been used as an experimental model for multiple sclerosis and that induction of HO-1 pathway suppress neuro-inflammation in EAE^23^. Induction of HO-1 also suppressed IFN-γ and TNF-α responses of CNS-infiltrating T cells. Suppression of Th1 responses by HO-1 was also reported in type 1 diabetes model in NOD mice^25^. Although modulation of Th17 responses by HO-1 in EAE was not analyzed in the previous report, it is likely that HO-1 suppresses Th17 responses as anti-inflammatory functions of HO-1 in non-eosinophilic asthma were associated with inhibition of Th17 responses^55^.

Several therapeutic strategies including injection of tolerogenic cells, recombinant proteins, monoclonal antibodies to inflammatory cytokines, pharmacological agents and oral tolerance have been explored in EAE^56^,^57^. However, long-term safety issues and particularly in pregnant and lactating women are of major concern with currently used therapies for MS^58^. Promising clinical results in relapsing-remitting multiple sclerosis prompted dissection of cellular and molecular mechanisms of action of IVIG in EAE^58^. We have recently reported that IVIG inhibits both Th1 and Th17 responses in EAE model^41^ and similar to HO-1 induction model of Chora *et al*.^23^, significantly inhibited CNS infiltration of T cells. Confirming the *in vitro* results, the protection rendered by IVIG in EAE was not associated with HO-1 induction. It was however not surprising given that endogenous expression of HO-1 had no consequence on EAE and daily injection of CoPP was required to induce HO-1 in mice and to protect from EAE^23^. Current data show that without activating HO-1, therapeutic benefits could be obtained in EAE. Thus, our results exemplify the multi-faceted mechanisms of IVIG to exert anti-inflammatory effects independent of HO-1 pathway. These results could be further consolidated by using HO-1-deficient mice. However, data from such mice would be difficult to interpret due to complex interactions of the HO-1 pathway products with various immune and non-immune cells.

IVIG products are not uniform and display variations with respect to formulation, stabilizing agents and source of plasma of healthy donors. Although these variations could influence the outcome of therapy of primary immunodeficiency patients, all IVIG products appear to have similar therapeutic benefits in autoimmune patients. Of note, when different IVIG preparations were examined for their effect on DC activation, endothelial functions, and Th17 differentiation and expansion, all tested IVIG preparations exerted similar effects^37^,^59^. In this report, we found that lack of induction of HO-1 by IVIG is not restricted to particular innate cell or IVIG preparation. In addition to Gamunex®, other three IVIG preparations (Sandoglobulin®, Tegeline® and Gammagard®) were also inefficient to induce HO-1. Thus, our data indicate that inability of IVIG to induce HO-1 is a universal phenomenon irrespective of preparations.

## Methods

### Human Cell culture

Buffy coats from the healthy donors were purchased from Centre Necker-Cabanel, Etablissement Français du Sang (EFS), Paris, France. Ethical committee permission was obtained for the use of buffy bags of healthy donors (Institut National de la Santé et de la Recherche-EFS ethical committee permission N°12/EFS/079) and experiments were performed in accordance with the approved guidelines of INSERM. Peripheral blood mononuclear cells (PBMCs) were purified from the buffy coats by density gradient centrifugation using Ficoll-paque PREMIUM (GE healthcare, France). CD14^+^ monocytes were isolated from PBMCs by positive selection with CD14 microbeads (Miltenyi Biotec, France). They were cultured for 6 days in RPMI-1640 medium plus 10% fetal calf serum containing GM-CSF (1000 IU/million cells) and IL-4 (500 IU/million cells) to obtain monocyte-derived DCs^60^.

### Murine macrophages

All animal studies were approved and performed according to the guidelines of Charles Darwin ethical committee for animal experimentation (Université Pierre et Marie Curie Paris) at the pathogen-free animal facility of Centre de Researche des Cordeliers, Paris. Murine peritoneal macrophages were extracted from C57BL/6J mice (purchased from Janvier Laboratories, France) by intraperitoneal lavage and cultured in Dubelcco’s Modified Eagle’s Medium supplemented with 1% penicillin, 1% streptomycin, 5% amino acids and 5% fetal bovine serum.

Murine RAW264.7 macrophages were maintained in Dubelcco’s Modified Eagle’s Medium supplemented with 1% penicillin, 1% streptomycin, 5% amino acids and 5% fetal bovine serum.

All cells were maintained at 37 °C in humidified air containing 5% CO_2_.

### Preparations of IVIG

Gamunex® (Grifols Bioscience, USA) was used throughout the study. In addition, Gammagard®, Sandoglobulin®, and Tegeline® were also used for *in vitro* experiments. They were dialysed against a large volume of PBS followed by RPMI-1640 medium at 4 °C for 18 hours to remove the stabilizing agents. IVIG was used at concentrations of 5, 10 or 15 mg/ml/0.5 million cells.

### Animals and EAE

All animal studies were approved and performed according to the guidelines of Charles Darwin ethical committee for animal experimentation (Université Pierre et Marie Curie Paris) at the pathogen-free animal facility of Centre de Researche des Cordeliers, Paris.

Ten-week old C57BL/6J mice (purchased from Janvier Laboratories, France) were injected intraperitoneal with CoPP 20 mg/kg. After 24 hours, liver and kidney were recovered and snap-frozen for western-blot analysis to check the HO-1 expression.

To induce EAE, C57BL/6J mice (10/group) were immunized with 200 μg MOG_35–55_ peptide emulsified in complete Freund’s adjuvant (1:1 by volume containing 800 μg of nonviable desiccated *Mycobacterium tuberculosis* H37Rv. In addition, 300 ng of pertussis toxin was given intravenously on the same day and 2 days later. Clinical signs of EAE were assessed daily based on the following scoring system: 0, no signs; 1, tail paresis; 2, hind limb paresis; 3, hind limb paralysis; 4, tetraplegia; and 5, moribund. From the day of the immunization until the peak of the disease (day 19), mice received daily intraperitoneal injections of 16 mg (0.8 g/kg) IVIG (Gamunex®). The control groups received equal volumes of 0.2 M glycine, the excipient of Gamunex®).

### Detection of HO-1 by Western blot

Human monocytes, DCs and RAW264.7 macrophages (0.5 million cells per ml) were treated with different IVIG preparations and with CoPP 25 μM, the activator of HO-1. After 24 hours, supernatants were removed and cells were lysed with a lysis buffer (20 mM dithiothreitol, 6% SDS, 0.25 M Tris, 10% glycerol and 10 mM Na Fluoride, pH = 6.8). In another set of experiments, following 24 hours treatment of monocytes with IVIG, LPS (10 ng/ml; Sigma-Aldrich, France) was added to the cells to stimulate the monocytes and to induce inflammatory cytokines. After 24 hours, supernatants were removed and cells were lysed.

Liver and kidneys from EAE mice at the peak of the disease (day 19 following induction of EAE) or from the mice injected with CoPP were lysed with the lysis buffer.

Proteins were separated by sodium dodecyl sulfate-polyacrylamide gel electrophoresis under reducing conditions and transferred to polyvinylidene fluoride membrane. HO-1 was detected using an anti-HO-1 rat monoclonal IgG (R&D Systems, France), a horseradish peroxidase-conjugated rabbit anti-rat IgG, and the enhanced chemiluminescence kit. β-actin was detected with a mouse anti-β-actin antibody (Sigma-Aldrich).

### Phenotype analysis of monocytes treated with IVIG

Peripheral blood monocytes from the healthy donors were stimulated with LPS for 30 min. They were then exposed to IVIG for 48 hours. The expression of CD80 and CD86 was analyzed by flow cytometry using PE-conjugated MAbs to CD80 and FITC-conjugated MAbs to CD86 (both from BD Biosciencies).

### Cytokine analysis

IL-6 in the cell-free culture supernatants was quantified by ELISA (Ready-SET-Go, eBioscience, France). The detection limit was 2 pg/ml.

### Measurement of NO production

Peritoneal macrophages from C57Bl/6J mice and RAW264.7 cells were treated with IVIG (10 mg/ml) for 24 hours. They were then exposed to either LPS alone or LPS and SnMP (25 μM; Frontier Scientific, USA) for additional 24 hours. Production of NO was evaluated by Griess method.

### Statistical analysis

Two-way analysis of variance (ANOVA) with Bonferroni’s post-test was used to analyze daily clinical score. One-way ANOVA was used to determine the statistical significance of the *in vitro* data. *P* value of less than 0.05 was considered significant.

## Additional Information

**How to cite this article**: Galeotti, C. *et al*. Heme oxygenase-1 is dispensable for the anti-inflammatory activity of intravenous immunoglobulin. *Sci. Rep*. **6**, 19592; doi: 10.1038/srep19592 (2016).

## Supplementary Material

Supplementary Information
