# Supplementary material for: Heme oxygenase-1 is dispensable for the anti-inflammatory activity of intravenous immunoglobulin
Source: Sci Rep. 2016 Jan 22;6:19592. doi: 10.1038/srep19592 (PMC4726216; doi:10.1038/srep19592)
Supplement: Supplementary Information [file srep19592-s1.pdf]

Supplementary Information

Heme oxygenase-1 is dispensable for the anti-inflammatory activity of intravenous immunoglobulin

Caroline Galeotti [1,2,3,4], Pushpa Hegde[1,3], Mrinmoy Das[1,2,3], Emmanuel Stephen-Victor[1,2,3], Fernando Canale[1,3], Marcos Muñoz[1,3], Varun K Sharma[1,3], Jordan D Dimitrov[1,2,3,5], Srini V Kaveri[1,2,3,5,6] and Jagadeesh Bayry[1,2,3,5,6]

[1] Institut National de la Santé et de la Recherche Médicale Unité 1138, Paris, F-75006, France

[2] Sorbonne Universités, UPMC Univ Paris 06, UMR S 1138, Paris, F-75006, France

[3] Centre de Recherche des Cordeliers, Equipe - Immunopathology and therapeutic immunointervention, Paris, F-75006, France

[4] Department of Pediatric Rheumatology, National Referral Centre of Auto-inflammatory Diseases, CHU de Bicêtre, le Kremlin Bicêtre, F-94270, France

[5] Université Paris Descartes, Sorbonne Paris Cité, UMR S 1138, Paris, F-75006, France

[6] International Associated Laboratory IMPACT (Institut National de la Santé et de la Recherche Médicale, France - Indian council of Medical Research, India), National Institute of Immunohaematology, Mumbai, 400012, India

Correspondence to: Jagadeesh Bayry or Srini V Kaveri, Institut National de la Santé et de la Recherche Médicale Unité 1138, Centre de Recherche des Cordeliers, 15 rue de l'Ecole de Médicine, Paris, F-75006, France. Tel: 00 33 1 44 27 82 03 ; Fax: 00 33 1 44 27 81 94

E-mail: jagadeesh.bayry@crc.jussieu.fr or srini.kaveri@crc.jussieu.fr

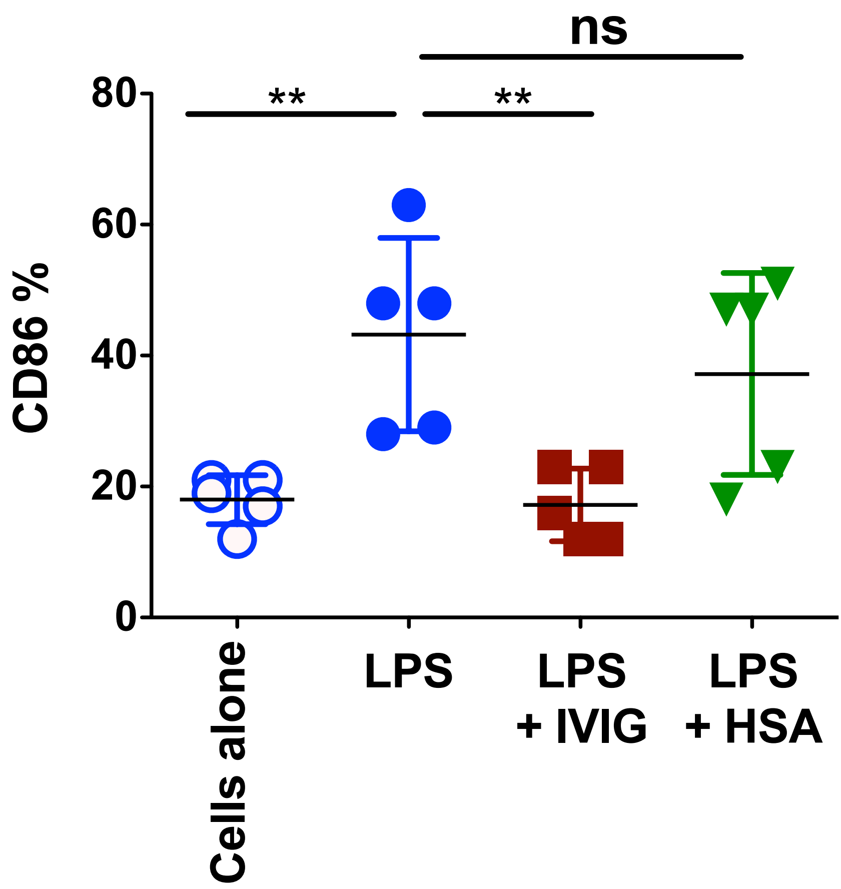
ns
**
**
80
60
40
20
0
CD86 %
Cells alone
LPS
LPS
+ IVIG
LPS
+ HSA

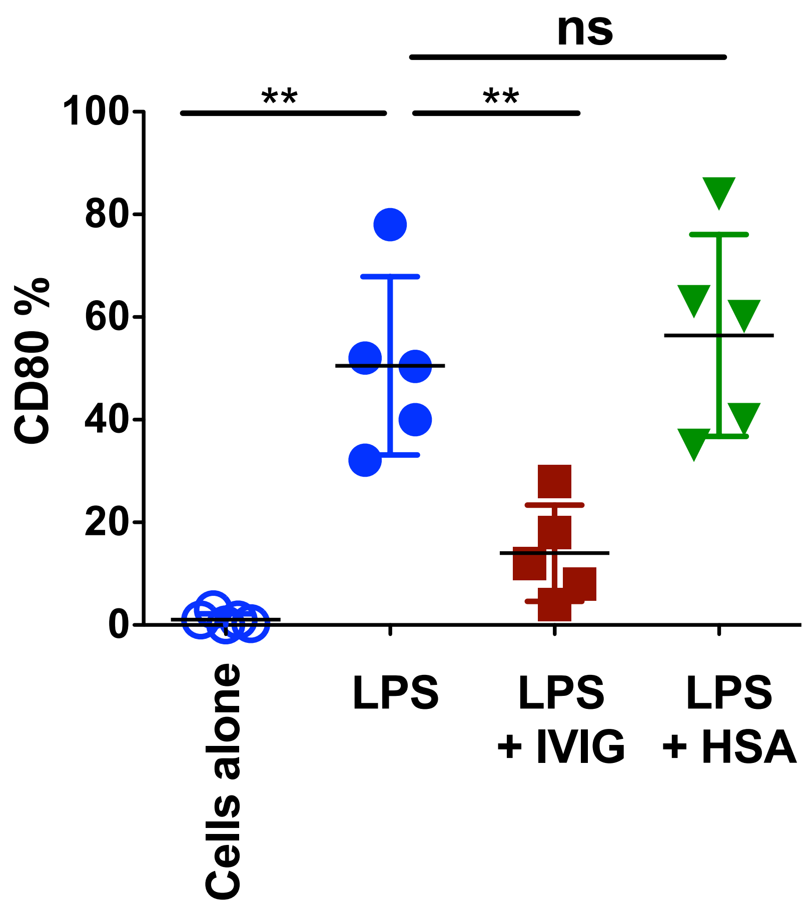
ns
**
**
100
80
60
40
20
0
CD80 %
Cells alone
LPS
LPS
+ IVIG
LPS
+ HSA

Supplementary Figure S1. IVIG inhibits LPS-mediated activation of monocytes as analyzed by phenotype. Peripheral blood monocytes were either cultured alone (Cells alone) or stimulated with LPS for 48 hours. In some conditions, after 30 min LPS stimulation, cells were treated with either IVIG or equimolar concentrations of human serum albumin (HSA) for 48 hours. The expression of CD86 and CD80 was analyzed by flow cytometry (n=5 donors). **p < 0.01, One-way ANOVA and ns, not significant.

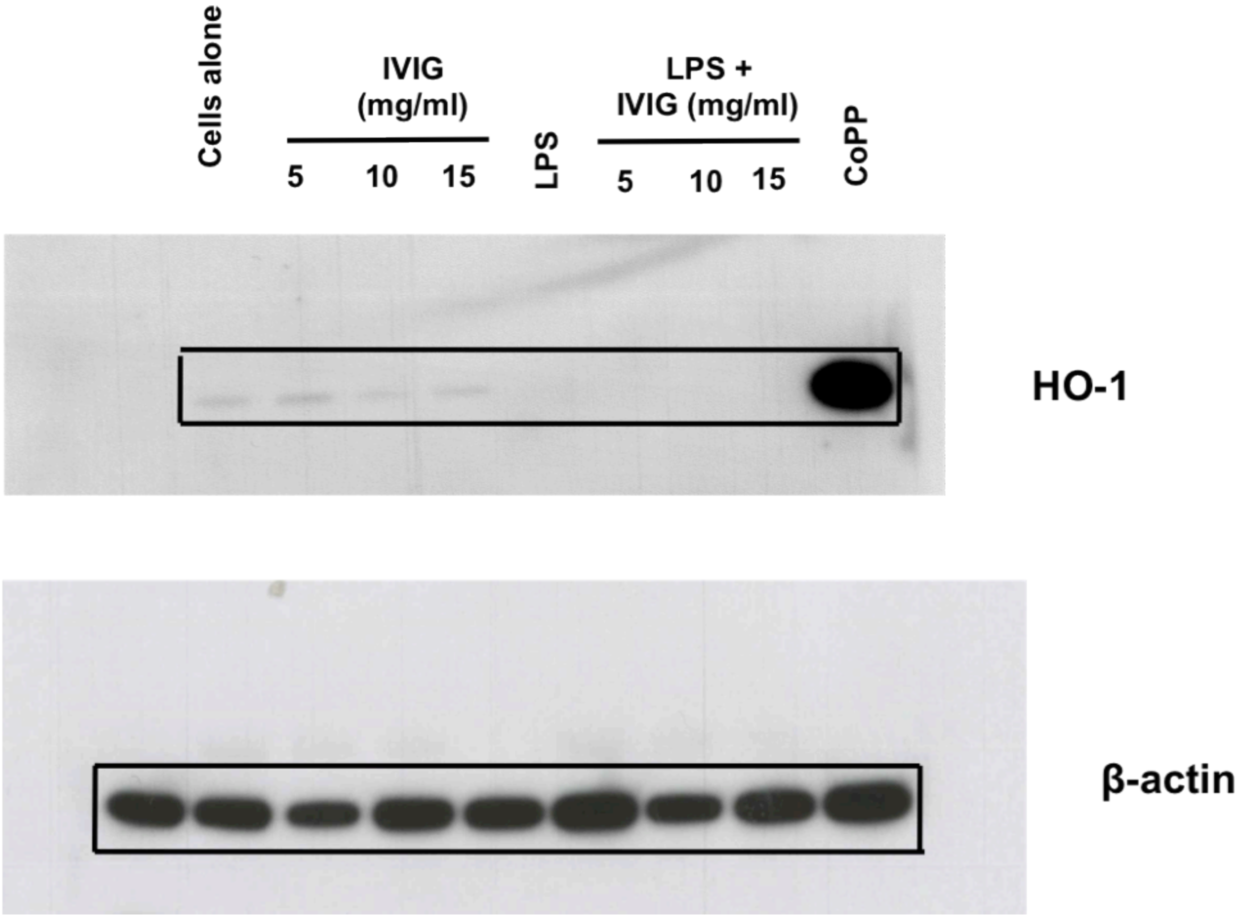

Supplementary Figure S2. Original western blots for images used in Figure 1b. Black boxes indicate the specific bands used in the main figure.
